# Steering the energy with music: hermeneutic phenomenological study of user perspectives of music and music therapy for co-occurring ADHD and substance use problems

**DOI:** 10.1186/s13011-024-00594-x

**Published:** 2024-01-23

**Authors:** Claire M. Ghetti, Steinar Hjelmbrekke, Katharina Morken, Therese Dahl, Brynjulf Stige

**Affiliations:** 1 GAMUT—The Grieg Academy Music Therapy Research Centre, The Grieg Academy—Department of Music, University of Bergen, 5020 Bergen, Postbox 7805, Norway; 2 Department of Addiction Medicine, Haukeland University Hospital, Bergen, Norway; 3 Department of Clinical Psychology, University of Bergen, Bergen, Norway; 4 Albatrossen Aftercare Center (The Albatrossen Foundation), Bergen, Norway; 5 Polyfon Knowledge Cluster for Music Therapy, Bergen, Norway

**Keywords:** User perspectives, Music therapy, ADHD, Substance use, Dual diagnosis, Motivation, Mastery, Social belonging, Qualitative research

## Abstract

**Background:**

User perspectives and involvement are crucial for improving substance use treatment service provision. First-hand accounts provide rich perspectives on how users experience change within therapeutic approaches like music therapy. People with substance use problems have a higher incidence of experiencing challenges with impulsivity, hyperactivity and inattention. Such challenges can negatively affect social functioning and outcomes of substance use treatment. Music therapy can offer people a means to regulate emotions and facilitate social relationships. There is a lack of research on user perspectives of music therapy in substance use treatment, and we could identify no studies that explore user perspectives of music therapy for adults with substance use problems and co-occurring impulsivity, hyperactivity and inattention.

**Methods:**

The aim of this phenomenological study was to center the voices of people living with co-occurring attention deficit hyperactivity disorder (ADHD) and substance use disorder (SUD) to understand how they experience music and music therapy in their process of recovery. We used a hermeneutic phenomenological approach to qualitative analysis of transcripts from in-depth interviews with 8 adult service users from a Norwegian substance use treatment facility.

**Results:**

Our main finding was that music and music therapy enabled experiences of motivation and mastery that ultimately afforded social belonging. The participants demonstrated detailed and nuanced understanding of how they use music to steer the energy and restlessness that are characteristic of ADHD, to change mood, and to shift negative thought patterns. These forms of music-centered regulation served as pre-requisites for more active and gratifying participation in social communities. For several participants, musicking offered a means of establishing drug-free identity and fellowship. The motivation and mastery experienced during musicking lowered the threshold for social engagement, and served as an incentive for continuing substance use treatment for some participants.

**Conclusions:**

The nuanced descriptions from our participants illustrate the importance of motivation, and how music therapy can contribute to motivation in substance use treatment. In particular, the context surrounding musicking, adaptations from the music therapist, and social affordances of such musicking contributed to pleasure, mastery, participation, development of identity and social belonging, which in interaction generated motivation.

**Supplementary Information:**

The online version contains supplementary material available at 10.1186/s13011-024-00594-x.

## Background

Substance use problems impact individuals, families, and communities in a myriad of ways; and treatment is complex and multifaceted. User perspectives and user involvement are crucial aspects of forming and improving treatment provision, and are particularly valued in a recovery orientation [1, 2]. Within the recovery model, change is not limited to symptom reduction but encompasses social and personal wellbeing [3, 4]. Understanding change from a recovery perspective has been especially important for people who experience co-occurring mental health and substance use challenges. When considering treatment approaches that take a recovery orientation, as is often the case for music therapy, it is important to gain rich perspectives on how users experience such services. Qualitative research of user perspectives enables a deeper examination of emotional experience framed in users’ own understandings and terminology [5] to answer questions of how and in what way participants experience change [6]. As experts by experience, service users have rich lived experiences of using music and music therapy in intentional ways that may contribute to social and personal wellbeing. Hearing and attending to user perspectives can beneficially inform both health service provision [2] and future research. In this study, we center the voices of people living with substance use problems who experience pervasive challenges with impulsivity, hyperactivity and inattention; to understand how they experience music and music therapy in their process of recovery.

### Co-occurring substance use problems and ADHD

People with substance use problems report more frequent challenges linked to impulsivity, hyperactivity and inattention (IHI) than people without substance use problems [7]. Impulsivity is considered an etiological pathway to the development of SUD [8]. People with higher levels of IHI are likely to have longer histories of substance use and greater mental distress [7]. Various conditions contribute to IHI and a portion of people who experience IHI are diagnosed with attention deficit hyperactivity disorder (ADHD), defined by ICD-11 as persistent “inattention and/or hyperactivity-impulsivity that has a direct negative impact on academic, occupational, or social functioning” [9]. Inattention consists of distractibility, problems with organization, and difficulty sustaining attention unless one is highly stimulated and receives frequent rewards (WHO, 2019). Prevalence rates for ADHD among adults with substance use problems are higher than among the general population, ranging from 7.6 to 32.6% with higher rates reported in the Nordic countries, and in particular Norway [10].

People who were diagnosed with ADHD as children demonstrate higher risk of eventually experiencing dependence on nicotine, alcohol, marijuana, or illicit substances [11]. People with ADHD are often the target of stigmatization and social avoidance beginning early in life [12] and can feel out of sync with those around them [13]. People with SUD experience social stigma and self-stigma that can negatively impact relationships, employment, health and the process of treatment [14]. Challenges with social functioning and insecure attachment strategies are common among adults with ADHD and SUD and may compromise the ability to form close relationships [15, 16]. As IHI is associated with increased likelihood of developing substance use problems and is also linked to higher likelihood of discontinuing substance use treatment, addressing IHI is an important focus for prevention and substance use treatment [7, 17].

### Treatment considerations for co-occurring ADHD and SUD

Research on user perspectives suggests that some substance users with ADHD perceive substance use as helpful for their emotional functioning, with benefits outweighting drawbacks, which has implications for motivation to seek substance use treatment [5]. It is theorized that the relationship between ADHD and substance use problems can be explained by an interplay among factors including a desire to self-medicate, disinhibition and comorbidity [18]. Underlying mood instability may motivate substance users to take substances in order to regulate mood and cope with restlessness [18]. Traditional substance use treatment approaches may not be a natural fit, since adults with ADHD and SUD demonstrate more frequent dropout, relapse and less positive outcomes in treatment than those without ADHD [19–21]. People with ADHD and SUD report moderate improvement in IHI symptoms with pharmacotherapy but also concomitant undesireable side effects [19]. Non-pharmacological approaches that help users identify constructive ways to manage feelings, identify destructive patterns of thought and behavior, and foster motivation for developing healthier lifestyles are indicated [18]. Approaches that are resource-oriented, active, gratifying and multimodal may be particularly helpful for engaging people with ADHD [18, 19, 22].

### Music therapy and substance use treatment

Music therapy can be defined as a practice facilitated by a music therapist consisting of “situated health musicking^1^ in a planned process of collaboration between client and therapist” [24, p.200]. In music therapy, therapeutic change is enabled by engagement in musical experiences and by the relationships that develop through those experiences [25]. The music therapy practice studied in this article is situated within a treatment system that follows national guidelines from the Norwegian Directorate of Health. Such guidelines recommend that substance users who wish should be offered music therapy as part of their treatment, provided by a music therapist with approved education^2^.

Synthesis of qualitative research supports use of music therapy for adults with substance use problems to facilitate group interaction, emotional expression, and skill development; and to improve quality of life [26]. Experimental research suggests that music therapy leads to moderate reduction in substance use craving and increases motivation for treatment and change in adults with SUD [27]. Research exploring resource-oriented approaches to music therapy and from a broader range of cultural and treatment contexts is needed [26–28]. There is a notable lack of research on user perspectives [29] and the specific needs of people with co-occuring mental health challenges and SUD.

### Music therapy and ADHD

Researchers have explored what music therapy affords children and adolescents with ADHD [30], but such research has been completed outside the context of SUD. We could identify no published studies that specifically explored the use of music therapy for people with ADHD and substance use problems. We therefore rely on the aforementioned literature on benefits of music therapy for people with SUD, in combination with research on music therapy for people with co-occurring substance use and mental health problems that suggests that music therapy can increase motivation for treatment and change [27, 31], regulate emotion [26, 32], reduce hyperarousal and improve attention [32], and reduce substance craving [27].

### Rationale for the study

User perspectives and user involvement form a crucial component of service development in a recovery-oriented paradigm. It is necessary to gain a nuanced understanding of user experiences of music and music therapy for those with co-occuring ADHD and SUD in order to meaningfully inform service delivery and future research. Music therapy may provide unique therapeutic opportunities for those who experience challenges with restlessness and attention, by supporting the regulation of emotions and facilitating social relationships. Research indicates that music therapy increases motivation for treatment and reduces substance craving [27], but we lack detailed descriptions of how users with ADHD and SUD use music in their daily lives and what music therapy affords them. There is a lack of research on non-pharmacological treatment of people with ADHD and SUD [19] and we could identify no studies that explore how people with ADHD and substance use problems experience music and music therapy. Therefore, the aim of this study was to explore how adults with co-occurring ADHD and substance use problems experience music and music therapy, and what this affords in terms of their health and their experience of ADHD.

## Methods

### Study design

We conducted a hermeneutic phenomenological study using in-depth interviews to explore experiences of music and music therapy among adults with substance use problems and ADHD. Hermeneutic phenomenology is derived from the philosophy of Heidegger, Gadamer and Ricoeur among others, and establishes a fusion of hermeneutics and phenomenology as a way of understanding situated human experience [33]. We adopt a constructionist epistomology for this research, viewing our findings as knowledge constructed between the participants and researchers, influenced by social and historical contexts [34]. We used the EPICURE evaluation agenda for qualitative research to guide our reflexive process during conduct and reporting of the research [35].

### Participants, setting and music therapy offer

We recruited 8 participants in the period 2017 to 2019 from a substance use treatment facility located in Norway that practiced gender-specific treatment of substance use disorders and offered both in-patient and out-patient services. Gender-specific treatment entailed separate residential units for men and for women, with specific therapeutic groups set aside exclusively for women or men^3^. At the time of the study, the facility was the only institution in the region offering music therapy to people with co-occurring substance use disorders and ADHD. Diagnosis of SUD occurred during either in-patient or out-patient assessment by the facility. Original inclusion criteria consisted of the following: (1) diagnosis of substance use disorder according to ICD-10^4^ (WHO, 2019), codes F10-16 (with exclusion of caffeine F15) and F18-F19; (2) diagnosis of ADHD for at least 3 months; and (3) participated in music therapy for at least two months during treatment. During recruitment, it became necessary to adjust down the requirement for length of experience of music therapy, due to trends in actual treatment length. Participant demographic characteristics are provided in Table 1.

**Table 1:** Participant characteristics

	Age (years)	Pharmacological treatment^a^	Length in music therapy	Time of ADHD diagnosis	Phase of substance use treatment^b^
A	40+	Yes	Over 1 year	Over 2 years	Completed treatment
B	26–40	No	3–6 m	Over 2 years	Inpatient long-term rehabilitation
C	40+	Yes	Over 1 year	Over 2 years	Outpatient
D	18–25	No	Over 1 year	Over 2 years	Outpatient
E	40+	Yes	3–6 m	Over 2 years	Inpatient long-term rehabilitation
F	40+	Yes	Over 1 year	Over 2 years	Outpatient
G	26–40	Yes	Less than 3 m	3–6 m	Inpatient long-term rehabilitation
H	40+	Yes	3–6 m	Over 2 years	Inpatient long-term rehabilitation

Note: Gender is omitted to assure anonymity

^a^Pharmacological treatment for ADHD

^b^Average duration of inpatient long-term rehabilitation is 3 months

Participants were at different stages of engagement in music therapy and received services from one of the four master’s-prepared music therapists at the facility. The music therapists had theoretical orientations that reflected common elements of the Norwegian music therapy tradition. Namely, music therapy offers were built around participant interests, aims and needs, with a strong focus on resources and mastery, where facilitating social and community participation were central [36]. In keeping with resource-oriented and recovery-oriented approaches, the music therapists worked to help support participants’ inherent resources and to develop a collaborative therapeutic relation based on equality and reciprocity in order to support participants’ processes of personal and social recovery [37, 38]. Founded in relational processes, participants engaged in musicking with peers and with the music therapist, in individual and group formats that included elements of songwriting, discussion around song texts, learning an instrument, listening to and discussing music, music-assisted relaxation, playing instruments together (often in rock idiom instrumentation), music improvisation, music production using digital audio workstations and performances. This broad variety of music therapy methods assured that participants could engage in musicking in alignment with their preferences and needs, and could choose individual or group formats according to what felt most comfortable to them. Verbal processing was also present in music therapy sessions to varying degrees. Both the frequency and duration of music therapy engagement varied across the participants.

### Data collection

In-depth interviews were conducted with participants, using a semi-structured interview guide (Appendix). Certain demographic information was obtained prior to starting the open-ended questions. The interviews were conducted by TD (4 participants), SH (3 participants) and another music therapist at the facility (1 participant), assuring that the interviewer did not have a dual role (such as treating music therapist) with the interviewee. Interviews varied from approximately 30–60 min each and were audio-recorded with a voice recorder. Interviews were transcribed verbatim by SH (3 participants) or by a research assistant (5 participants).

### Data analysis

CG analyzed transcripts using qualitative analysis from a hermeneutic phenomenological perspective [39, 40], attempting to make meaning out of the participants’ meaning making of their lived experiences of music and music therapy. The analysis process began with reading each transcript through to get a sense of it, starting with the most “rich” interview and underlining segments of text that related to participants’ experiences of the phenomena of interest. CG then coded segments with use of in vivo coding [41] to create descriptive comments, maintaining participants’ own wording where possible. After reading through the transcript again, CG added conceptual comments to help enrich understanding of the phenomenon, adjusted/expanded codes to represent all discrete experiences, organized codes relevant to the research question into sub-themes and themes, and arranged these into a table. Themes were organized into domains within the table, and rich illustrative quotations were inserted for each theme. After analyzing the first interview transcript, CG analyzed within and across the remaining interviews and integrated their sub-themes, themes (if any new), and quotations into the main table.

Two co-researchers (SH & KM) then read through the transcripts and coding table and identified places where they concurred or disagreed with the themes and sub-themes. Consistent with a hermeneutic phenomenological approach, SH, KM and CG engaged in dialogues to use reflexivity and interpretation as means of more deeply understanding each participant’s experiential material within their unique context. All phases of the qualitative analysis were completed in Norwegian to match participants’ language use, and the findings were translated to English during generation of the manuscript.

## Results

During analysis it became clear that we needed to understand our participants’ experiences of living with ADHD, before we could understand the role that music and music therapy have in such experiences. Thus, we organize our findings into two domains: (1) experiences of ADHD, and (2) experiences of music and music therapy; where the first domain provides a foundation for contextualizing the second. A synthesized description of the participants’ experiences is included within each theme, organized by sub-theme and substantiated with quotations from the participants. Where possible, we have retained participants’ wording in sub-themes and themes, albeit as translations from Norwegian. We also distinguish between experiences described by one, several, all or no participants.

### Experiences of ADHD

Living with ADHD is experienced by participants to varying degrees as: being steered by energy, being pressured to be like others, giving rise to self-medicating, and requiring a life-long process of coping.

#### Steered by energy

Several participants describe a tight connection between body sensations, feelings and thoughts and that their whole experience is “steered by energy.” Emotions are expressed through the body, which then impacts thoughts. Participant B describes first experiencing restlessness as manifested in the body, and thereafter being flooded with thoughts, but has the ability to regain balance by listening to trance music. Experiencing a bodily manifestation of emotion was present in childhood for Participant H:

*When I was a boy, I ran all the time. You don’t manage to express depression, anxiety, restlessness, or anything at all. You say maybe you have a bit of a stomachache. You don’t have words for it*. (H)

The restlessness that one experiences is coupled with challenges in concentration. Participants describe “taking in so many unnecessary things”, trying to process everything simultaneously and in so doing, being at risk of missing something important (B).

*To concentrate on what one should concentrate on, when you need to concentrate, instead of your head constantly spinning and going on all sorts of things that it should not do there and then. Concentration, restlessness, nervosity, they are quite tiring to live with*… (G).

Participant G acknowledges that they^5^ have learned to live with this challenge in focusing attention, but it comes with a cost. Difficulties with concentration and following-through have also led some participants to struggle in making it through school and/or holding jobs, “It has been a struggle the whole way. You begin many different things, but I never manage to complete any of them” (G).

For some, the experience of restlessness is tied to feelings of self-worth,

*It can give low self-esteem and you can get anxiety, and somehow miss a little belief in yourself. And lots of impulsivity, that makes things quite difficult*. (E)

while others experience restlessness and aggressivity (D). Feelings of frustration often led to giving up, “…if frustrations arise or something like that, then it is much easier to give up on those things” (D). Some participants (A, C, G) felt a sense of clarity when they were diagnosed with ADHD, as Participant G describes, “all the signs and all I had struggled with my whole life, and why I struggled with it.”

#### Pressured to be like others

Four participants realized early in life that they were not like others of their age. Other people pointed out that they could not sit still and did not behave “properly” (C, E, F).

*And the same in elementary school, it was just misery and yelling and howling and screaming that you did not manage to behave properly. [I] was a bit impulsive also*. (C)

For Participant F, those impressions still “hang there” today:

*ADHD, it’s like you’re not like everyone else, right, sitting and drinking coffee. You are a bit too active, you have to do things…I can’t last long relaxing and being social for too long, I think it’s tiring*. (F)

One could feel fine while alone, but then be forced to put “brakes on” oneself when in social settings:

*…I have put so many brakes on myself, and it is not right to put so many brakes on yourself, but it becomes a part of the whole. And it’s tiring. And it becomes a bad habit in the end, because you have to be like everyone else.* (F)

Participant F experiences this self-censure as being placed in a “pen” since one is forced to behave differently than how one really is. A challenge arises when participants have to do things, such as go to school or learn things, on the premises of those who do not have ADHD:

*…then I struggle with that setting…but to take a practical job in construction, or organize other things, I have no problem with that. I do not think there was a problem in the Stone Age with ADHD*. (H)

#### A reason for self-medicating

Two participants (G, H) described turning to substance use as a way to cope with feelings of restlessness in their everyday lives.

*So when I started taking drugs and such, it was just like, you finally got peace in a way, you know? Finally got your mind to relax, finally got your body to calm down, kind of. It was just that you always searched for something that was missing, you know. Just as if something fell into place then*. (G)

Participant D experienced that their tendency towards aggression was lessened when using marijuana. This self-medicating approach that worked effectively for reducing aggression was then the source of Participant D getting “burned” by a system that punishes such activity. For another participant it was hash, alcohol and amphetamines that brought a sense of balance amid inner restlessness, but also undesirable legal consequences:

*…but if one is convicted many times for taking that medicine, the closest you can come to treatment for your diagnosis, to alleviate the symptoms, to be able to do something artistically creative, it stings a little*. (H)

Since substances enabled some participants to experience a feeling of “peace,” of relaxing and unwinding, it was easy to start using drugs more intensely “than one should” (G).

#### A lifelong process of coping

As participants get older, their experience of ADHD changes: outwardly apparent restlessness, hyperactivity and in some cases, aggression has transformed to inner restlessness.

*I was much more outwardly aggressive before, much sharper on the edges, and now I notice that it has become much more on the inside, in a way. Now it’s a little more inner restlessness.* (D)

An experience of “inward chaos” continues for Participant C, who also experiences ongoing problems with impulse control. For Participant C, ADHD medicine has helped with several aspects, but the inner restlessness continues.

Several participants express a bitterness over not having received an ADHD diagnosis or accompanying support earlier in life. They wonder how things could have been different:

*So a little bitter that this was not caught when I was little and that I could have gotten help and maybe been medicated then, too. And gladly would have avoided having the bad start to life that I had*. (G)

When Participant C read the report from being assessed for ADHD as an adult, they experienced “aha!” moments where things made sense, but also irritation that no one had intervened during school age. Participant C thinks that if they had received ADHD medicine as a child, their whole life situation would have been different.

At the same time that participants acknowledge the long-lasting challenges of ADHD, they also report possessing strengths including keen concentration and determination when highly motivated. For Participant H, having ADHD means that they have abilities that others do not:

*I do not look at it as a handicap, I look more at people who don’t have ADHD as a handicap, who can’t manage to have seven balls in the air at the same time. When we who have ADHD talk to each other, we have no problem understanding each other or remaining connected. It’s those who don’t have ADHD who can’t keep up. They are the ones who are slow (laughs)*. (H)

Other strengths include an ability to improvise, owing to challenges with planning ahead, and speaking spontaneously and earnestly. Participant H feels that ADHD only becomes a problem when one has to operate on the premises of those who do not have ADHD.

The participants have developed various strategies and tools to manage challenges related to ADHD. Several participants (A, C, E, F, G, H) report that ADHD medication is helpful in a variety of ways including for improving planning ahead, memory, and impulse control. However, inner restlessness persists despite the medication. Other strategies that help participants include exercise, learning to pause and think through things before acting, and using music to steer one’s energy. One learns to adapt themselves to living with ADHD, but it is not something one will ever “be rid of” (G).

### Experiences of music and music therapy^6^

Prior to engaging in music therapy, participants had engaged in music in various ways: playing an instrument, learning to read music, working with music production, or listening to music throughout the day. Some had always wanted to learn an instrument, but never managed it on their own. Others had a history of playing in bands, touring and releasing albums. Regarding music therapy, some participants preferred individual sessions where they could try things out in a different way than in the group, or could more closely have their music preferences met. Other participants preferred groups, which afforded more diverse social aspects and musical experiences. Participants’ experiences of music and music therapy can be understood within five themes: regulation, motivation, mastery, social belonging, and self-development.

#### Regulation

Participants experience music and music therapy as an important means of regulating several key features of their existence: energy, feelings, concentration, and thoughts. These features are tightly related to their experiences of both ADHD and substance use and are essential aspects of their daily lives.

**Energy**. Music is experienced as a flexible tool for “steering” the energy and restlessness that characterizes ADHD. Participant B experiences music as a “remote control” that can control all aspects of their life:

*Can either turn up the volume, fast forward, rewind, pause. It is a universal control, quite simply. And I don’t understand why there isn’t more of it in treatment places here, music therapy*. (B)

Participant B experiences music as a tool that controls all other tools and resources, such as “creativity and even sleep,” and has done so since childhood. As such, music is indispensable in Participant B’s life:

*Music for me is just a journey, in this journey here on earth that allows me to control an energy within me that I have struggled with my whole life– to steer– and that is probably the essence of music, that you can use it for absolutely everything in life. All phases, all feelings, all thoughts*. (B)

Other participants experience music as a way to “get out energy” (D, F) or calm down, depending upon what kind of music is used (C, F). Some participants experience the ability to control energy most strongly when playing instruments, such as guitar (F), while others feel it while listening to music during other activities. Participants express a refined understanding of what particular types of music have either calming or energizing effects for them. Some participants experience that loud music calms them down (C). The symptoms of ADHD make it challenging to achieve a sense of calm, and participants reported a rare experience of calmness through music:

*…music therapy has been one such thing where I have managed to find the calmness that probably ordinary people do in many things, in a way. Which, of course, I don’t manage to do with many things*. (G)

The energy participants feel is tightly related to feelings and thoughts.

**Feelings**. Participants describe several ways that music and music therapy are tools for working with feelings: expressing feelings, expressing oneself to other people, and coming in contact and exploring emotions.

*It’s about expressing oneself emotionally in front of other people, not just for yourself. It’s that bit– it’s kind of a therapeutic thing. And I do that best with music*. (H)

Participant H feels that as humans we want to have a word to describe each emotion, but that there are no words for many emotions. Instead, music may be a way to express those emotions (H). Participants use music intentionally to move out of a bad mood, to feel cozy, and to have good feelings without having to use drugs.

*You get something– a focus that is positive and it helps in terms of addiction. You get good feelings without using drugs*. (F)

Music therapy offers a means for participants to experience good feelings without having to use drugs.

**Concentration**. Music affords the participants a concrete means of promoting attention. Participants experience an ability to concentrate when engaging in music and music therapy that they do not otherwise experience. Participant B has a nuanced understanding of how music helps with concentration and describes the process they used for concentrating on schoolwork earlier in life. B would tap a foot along with the beat of the music, creating a kind of framework for the energy:

*Following the beat…what lies behind there is that it kept me occupied…that little switch that makes us have a deficit in attention became saturated and then I could concentrate*. (B)

With music playing through headphones, B could sit calmly in class and concentrate on work. Participant C becomes calm when concentrating on playing or singing and can even detach from the typical restlessness of mind and body. Participant E finds singing to be particularly helpful in holding concentration as E focuses on the melody and expressing the lyrics. Participant H also experiences the ability to hold and express a thought through music:

*Even though I don’t speak very well, hop from one to another thought, in music we can hold a thought or an expression…[In the music], I have a common thread*. (H)

Playing music provides an opportunity to practice concentrating and staying focused (H), which can enable one to “be in the moment.” Engaging in music enables participants to shift focus in a positive direction, which is experienced as freeing (F). By “being in the moment” in music, participants experience that time just “flies by”, including time in substance use treatment. Participants who play in band^7^ notice that their concentration is particularly sharpened when playing with others (F, G). Playing together also enables the group members to enjoy a shared focus, which is perceived as both enjoyable and enriching.

Participants experience that the music therapist’s ability to adapt music processes to the needs and abilities of those in music therapy helps lower the threshold for concentration and engagement. Several participants tried to take up instruments or learn about music earlier in life, but gave up when it became too difficult (B, C, D). Participant B experienced that the music therapist mentored B into a world of music programming that had otherwise been too difficult to access due to challenges with concentration.

**Thoughts**. Participants experience that music provides a means to shift thought patterns. They use music to shift to positive thoughts (F), to provide a contrast to heavy personal themes brought up in sessions with other substance use treatment personnel (A), or to shift away from thinking about substance use (D). Participants know how to use certain types of music at certain times and situations, to shift out of negative moods and cycles of thought. Participant D experiences that the process of music therapy strongly influences their thought patterns throughout the day, as it provides something constructive and future-oriented to focus on instead of perseverating on thoughts related to substance use.

*All the way home, mostly when I’ve had music therapy, then I sit and enjoy myself with the music [played or recorded in music therapy] and think “I learned this one. I can do a bit better on this one next time” and such. But on the days I haven’t had music therapy, I usually sit on the bus and just [think] “I want to go home now, I want to smoke* [hashish] *now*.” (D).

When leaving day treatment each day, Participant D leaves with “head raised” and feeling pleased with accomplishments in music therapy and therefore does not immediately begin thinking intensely about how they will get high. Participant D sometimes creates playlists the day before going to music therapy of songs that they want to try out in sessions. The act of creating the playlist constructively engages the participant so that they do not use drugs and replaces the intense desire for substances.

#### Motivation

Participants experience that music has provided motivation during several life stages, starting from when they were in school and extending to motivation to continue in substance use treatment. Participant B used music to concentrate in school and without music playing in headphones, lost desire to focus on schoolwork. Participants experienced music therapy as particularly motivating since the music therapist uses what one wants to do with music as a starting point, whether that is learning to sing or play an instrument or learning to digitally produce one’s own music. The majority of participants expressed a desire to spend more time in music therapy, as they were aware of what music affords them in terms of pleasure, a purpose and also a “tool” for various aspects of life.

Engagement in music therapy became a motivation for staying in treatment or opening up for other forms of treatment. Music therapy sessions were experienced as something positive and “cool” to engage in which helps one to thrive during lengthy treatment stays (G).

*I have enjoyed myself a lot with the music and have had a very nice time there, that has often given me new motivation and determination to continue in treatment.* (G)

The progress that Participant G made in music therapy became a source of motivation for continuing to engage in sessions and further develop oneself (G). Music was the primary motivating factor for Participant H to begin medication for ADHD, so that they could have better focus and concentration for working constructively with music.

The active aspect of music therapy was particularly appreciated, “…getting to talk to someone where you can move around, do what you want, too, instead of sitting with a therapist and just sitting there (Participant D).” It was important that instruments used in music therapy matched participants’ interests as well as physical and energy needs. Though Participant D had access to a guitar at home, drum set was a much better match for their physical impulses as it offered the chance to be more physically active and “hit something without hurting.” Participant H gains motivation from playing with others in music therapy and recognizes that they can accomplish things in music therapy that they cannot in other settings (H).

The low-threshold aspect of music therapy was experienced as important for motivation. The music therapist is able to adapt the group and individual sessions so that participants can engage actively regardless of their previous musical knowledge. Participants found this low-threshold aspect of the band group to be crucial to their engagement, as even participants with no prior experience of playing instruments could contribute in some way (C). The low-threshold aspect of music therapy services is reflected in the policy that people in treatment can use the music room independently when they desire. For example, Participant D took the long trip out to the treatment center during the winter holidays, just to use the room and play drums.

Continuity with the music therapist was also experienced as important for motivation. Participant F notes that people who have been in rehabilitation several times experience a high volume of therapists and workers. Participant F experiences frequently meeting new people as tiring and therefore appreciates seeing the same music therapists across phases of treatment.

#### Mastery

Motivation and mastery are tightly coupled in participants’ experiences of music therapy. Participants experience feelings of mastery and self-confidence when they recognize what they have managed to do in music therapy (E). Participants feel a sense of mastery and satisfaction when overcoming the challenge of learning something new, a type of persistence and payoff that has been infrequent because of ADHD (D). Living with ADHD, participants are used to beginning many things, but never managing to succeed with them except for music (G). This feeling of mastery is amplified when such successes are shared with friends:

*…pleasure from feelings of mastery…a lot of it has to do with a feeling of mastery, the feeling that you actually manage something. And in addition that you can go around later and put on a song when you are with some friends, say “I’ve learned this on drums” or “I know how to play this.” You get a little, “Oh, cool!”*. (D)

In such a way, the experience of mastery is re-lived when one listens back to a recording or shares such with others.

*No disrespect to therapists or anything like that, but when you just sit there and talk, and leave there and [think] “ok, I had a conversation, I am satisfied.” But when you have had music therapy, you sit back and talk and in addition, you have learned something, you have managed something new. So you leave there with head raised, and I actually prefer to go to music therapy rather than the usual therapist*. (D)

Managing something new in music therapy led to feelings of pride.

Adaptations and support from the music therapist helped participants experience increased determination and perseverance. Participant B viewed their music therapist as a “mentor” who helped adapt instruction in order to accommodate the participant’s challenges with concentration. Participant D experienced their music therapist as a motivator who reminded them, “you can do this.” In a context where many struggle with attending therapy sessions, Participant E shares that they have not missed a single music therapy session. Furthermore, when Participant E does not manage something satisfactorily in music therapy, they work on it independently in the evenings, which helps with mastery during the next session.

By their nature, the music therapy sessions offer positive challenges, for example, playing a solo in front of others, learning a new instrument or new techniques, or expressing feelings. Feelings of mastery came quicker when participants played instruments that matched their interests and needs. Participant D began with a motivation to learn many instruments, but eventually found that drumming was the most satisfying way to express the “foundational rhythm” that was in them. The learning process was a little easier, and motivation and determination where higher when participants engaged with instruments that matched their needs.

Challenges in music therapy can also be “negative” (H) and reinforce negative thought patterns and thus require support from the music therapist and peers to constructively manage. In general, it was a good feeling to master things, but participants could become easily irritated and sour when they did not manage something (G). Learning music is not always easy and patience can be a challenge, also in music therapy:

*It has, of course, been tiring and my patience isn’t always at its peak, but I’ve sort of gotten through it and tried to not have such high expectations of myself*. (G)

Participant G learned to take things step by step as ways to manage these challenges.

#### Social belonging

Music provides motivation to engage socially for several participants (A, B, F). Music therapy groups provided a reason to come out into the world and do something active instead of isolating at home. Participants experienced music as a means of engaging socially, which is a large part of its helpfulness for them. Some participants experience that playing music is healthpromoting even when one is alone (H), while others have found that it provides a crucial pathway out of depression, anxiety and social isolation (A).

*I lived with my mother and father and was actually scared to go out at all. I was at home a lot, so it was music that got me out after I finished here, so that was the motivation to, in any way, engage socially*. (A)

Playing in music groups was an essential motivator for Participant A to engage socially after they finished treatment. Other participants experienced that band groups were particularly helpful for social connection as they open up for various social and musical experiences. Participant F had been a musician for many years and took a long pause from playing until hearing about music therapy during the course of in-patient treatment. Participant F was highly motivated to play with others and joined the band group.

*It was very gratifying and social to play with others instead of just sitting and chatting and drinking coffee, yes, that you have a shared focus. I think it’s very, very fun and you feel good feelings*. (F)

Such groups were helpful both during in-patient treatment and in aftercare as they provided a new network and new friends, often without a focus on drugs. This drug-free fellowship enabled participants to have a shared focus on something positive and constructive, and offered a context where they did not have to talk about addiction. Such aspects made it easier to develop a satisfying sense of community.

*It was like that—the good shared things we could talk about without all that muck. Everyone had a sense of belonging, which we could talk about.* (B)

In previous social contexts, everything revolved around substance use, but in these music groups in treatment and aftercare, participants experienced social belonging where substance use was no longer a theme (A).

Discharge from treatment is often a highly vulnerable time, and participants experienced that music therapy and music engagement provided critical supports. Participants developed resources in music therapy that they could take further as strategies for the vulnerable time after discharge. Participant A learned enough music skills and developed enough self-confidence in music therapy during in-patient treatment that they dared to take contact with a music group at an aftercare program. Participant E planned to engage with music offers in their place of residence and participant G felt that the municipalities should be better equipped with low- and medium-threshold offers for people with various challenges. Participant B appreciated that the music therapist helped connect them with aftercare music services, as the “problems are not in treatment, they are outside of it.”

#### Self-development

Participants’ experiences of music therapy reflect themes of self-exploration, evidence of self-development, and development of new identity. Participants experience playing music as a process of self-exploration, particularly through improvisation.

*Because I wanted to find out what music would come out of me if I began making music totally openly…I just improvised, heard what the guitar did…came in contact with myself, with my own soul*. (H)

Participant D experiences becoming a bit more “outgoing” version of themselves when playing drums, which is enjoyable.

Over the course of music therapy, participants can perceive their development over time, both on personal and musical levels. Learning to sing, including just daring to try it, is perceived by Participant E as a satisfying example of personal development. Concrete musical qualities (like increased vocal range), serve as evidence of musical development (E). Participant G was excited to perceive development from very limited musical ability to where they are now and finds value in such development: “As long as I have a potential for development in it, that it doesn’t stop and stagnate, then I think it is nice and fun.” Participant G wants to continue to “develop” themselves, not in order to play in a band or for others, but to be able to sit and play guitar to relax and find peace.

Participants found that music therapy gave them an opportunity to develop and practice a new identity, one that coupled music with sobriety.

*Yes, to couple music and intoxication together is to make music a negative thing. Coupling music and sobriety, which is a constructive process, that is more what I do, a part of my identity, to remove substance use from my identity, beyond myself and for the world around me*. (H)

Participant H actively used music therapy as a way to build up a new identity. Developing new identity is one way that participants “break the pattern” of addiction (H). Participants find ways to use music as creative solutions for their problems, instead of just defaulting to the substance use milieu (A). Participant C felt that music therapy did not have particular influence on ADHD aside from developing a new interest, but at the same time acknowledged that playing an instrument at home could be a constructive thing. Playing instruments gives a new way to do things to “break out” of the pattern of substance use.

## Discussion

**Fig. 1:**
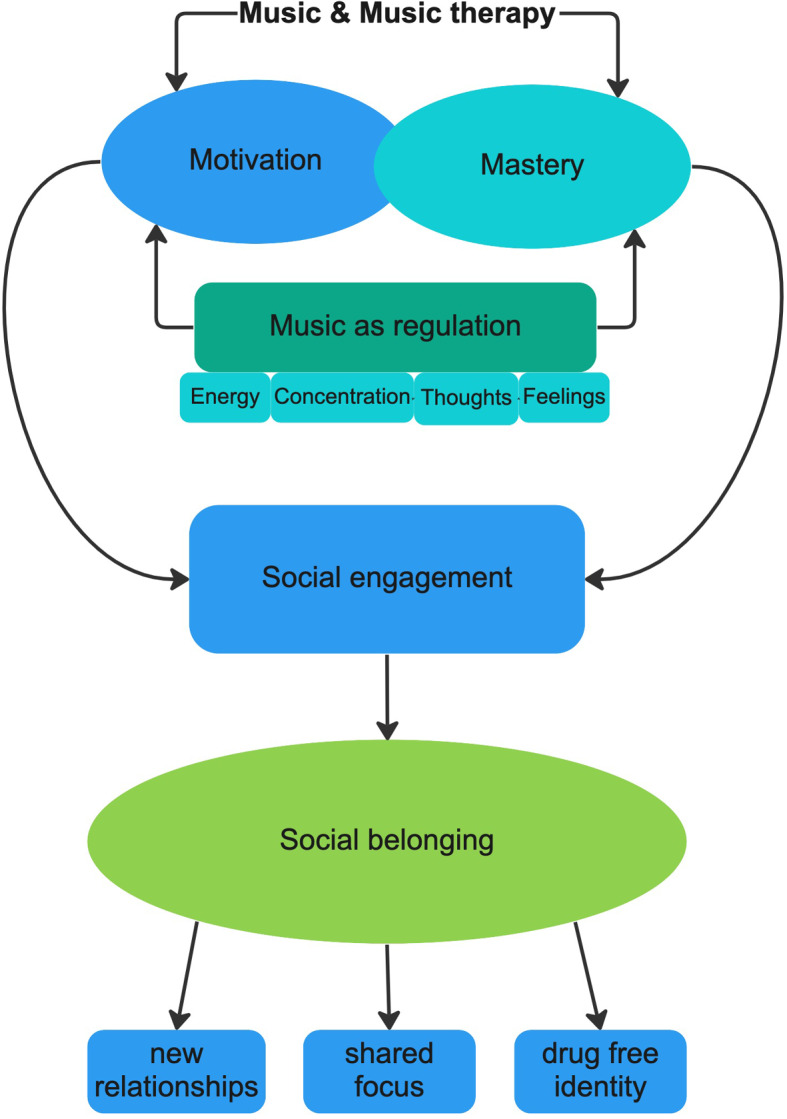
Contributions of music and music therapy to motivation, mastery and social belonging

This study centers the voices of people living with co-occurring ADHD and substance use problems, using a hermeneutic phenomenological approach to understand how they experience music and music therapy in their process of recovery. Through rich accounts of lived experience, the participants described how they engage with music and music therapy in intentional ways that promote both social and personal wellbeing. Our main finding was that music and music therapy enabled salient experiences of motivation and mastery that ultimately afforded social belonging (Fig. 1). The adults with ADHD interviewed in this study are resource rich. They demonstrated detailed and nuanced understanding of how they use music to steer the energy and restlessness that are characteristic of ADHD, to change mood, and to shift negative thought patterns. These forms of music-centered regulation served as pre-requisites for more active and gratifying participation in social communities. For several participants, musicking offered a means of self-development that enabled the establishment of drug-free identity and fellowship. The motivation and mastery experienced during musicking lowered the threshold for social engagement, and served as an incentive for continuing substance use treatment for some participants. We hope that the nuanced understandings described in this study help to capture the complex relational and contextual experiences that underlie the participants’ relationship to ADHD and to substance use. Our findings are particularly relevant for contexts reporting high correlation between substance use and ADHD, as has been noted in some Nordic countries [10].

Engaging in music, and particularly in group-oriented musicking, afforded participants experiences of social belonging. Music therapy groups provided a reason to venture out into the world and engage actively instead of self-isolating. Through such engagement, participants experienced social belonging through music, gaining new social relationships, and feelings of solidarity in a non-drug identity. For some participants, these social aspects were music therapy’s greatest affordance. People with ADHD can experience a fundamental feeling of being out of sync with those in their surroundings [13]. Participants in the current study found that playing music with others was a gratifying form of synchronized social engagement. Adaptations from the music therapist made it possible for participants of varying levels of musical competence to socially engage with others through musical synchrony. Facilitating social functioning is important for people with ADHD, as they are often the target of stigmatization, including social avoidance, that often begins early in life [12]. Our participants described how music therapy helped them develop skills that they could use to lower the threshold for social engagement during the critical period of aftercare. Music and music therapy provided a medium for experiencing a constructive and shared focus without the need for substances. Our finding that music and music therapy help provide motivation to engage socially is a highly significant one, since insecure attachment strategies common among adults with ADHD and SUD may compromise the ability to form close relationships [15, 16].

**Fig. 2:**
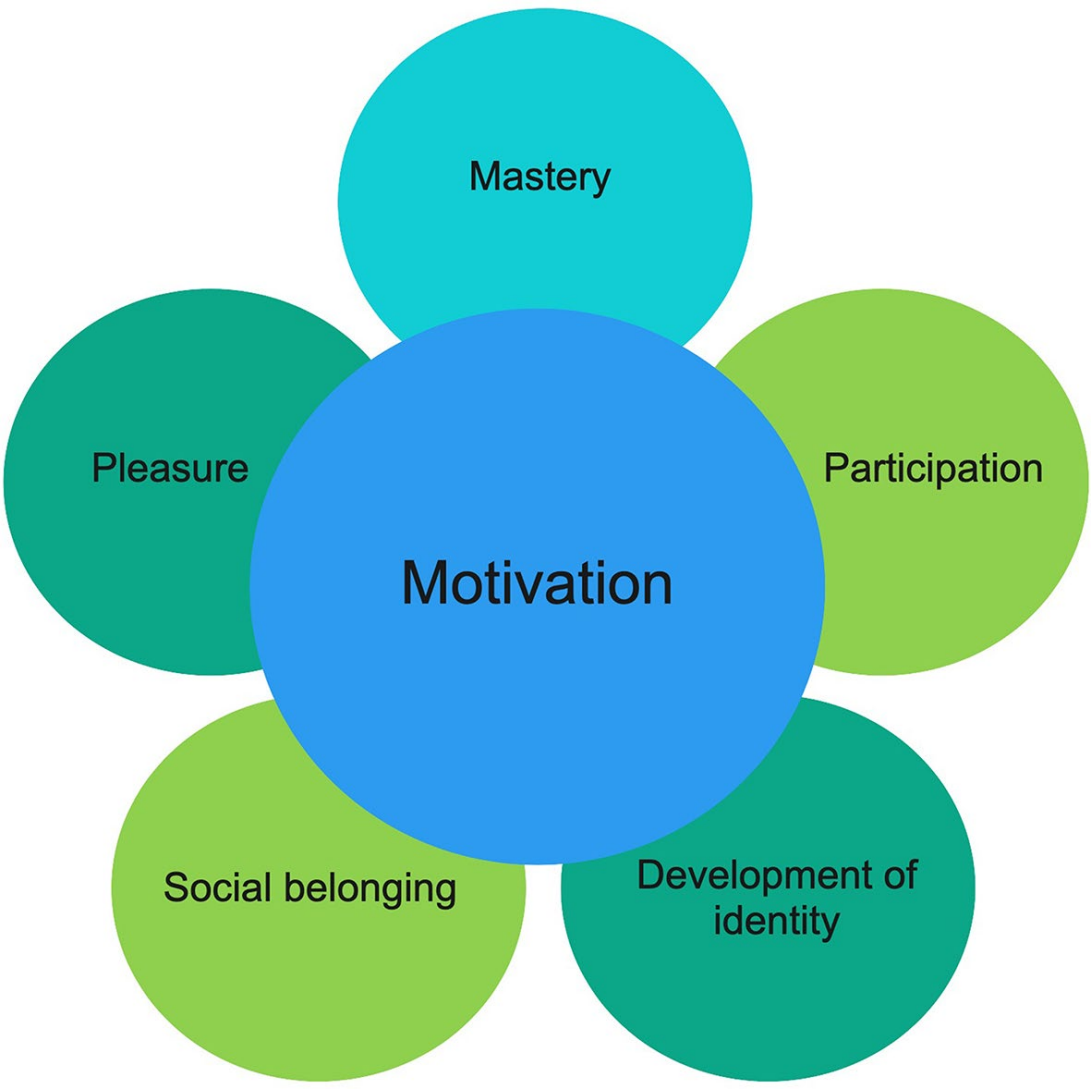
Aspects of music and music therapy that generate motivation

Motivation and mastery emerged as two vital and interlinked processes that active forms of music and music therapy afforded (for example, playing instruments, singing or engaging in music production). Within music therapy, the music therapist adapted interactions to participants’ needs, interests and strengths; and provided a level of motivational support and structure that helped participants experience mastery. Experiences of mastery then increased motivation for further engagement, which set the scene for additional experiences of mastery. The music therapists in this study use a resource-oriented form of music therapy where participants’ interests and self-determined goals serve as a starting point [37, 42]. The good feelings and sense of mastery that participants felt in music therapy played an important role in preventing further substance use and promoting a sense of well-being and satisfaction. Since high levels of mental distress are strongly correlated with IHI and risk for discontinuing substance use treatment [7], our findings have important implications for substance use treatment. Some participants articulated that engagement in music therapy motivated them to stay in treatment and to engage in other forms of treatment, such as verbal psychotherapy and pharmacotherapy. Music therapy enabled them to thrive during lengthy treatment stays and the progress and mastery experienced in music therapy became a source of motivation for continued engagement. Our findings confirm those of a recent meta-analysis of experimental research that suggests music therapy increases motivation for treatment among people with substance use disorders [27]. The nuanced descriptions from our participants illustrate the importance of motivation for them, and how music therapy can contribute to motivation in substance use treatment. In particular, the context surrounding musicking, adaptations from the music therapist, and social affordances of such musicking contributed to pleasure, mastery, participation, development of identity and social belonging, which in interaction generated motivation (Fig. 2). Such experiences reflected a health-promoting process that was simultaneously personal, social and situated. Participants engaged in music and music therapy in ways that developed constructive and healthy identities that offered a means to sustain them during integration into the community following treatment. Building new identities tied to musicking was one way that participants could “break the pattern” of addiction and experience gratifying aspects of self-development.

Findings from our study suggest that participants experience music and music therapy as means of regulation, motivation, mastery, social belonging and self-development. These experiences align with the processes of personal recovery outlined by the CHIME framework: connectedness, hope and optimism about the future, identity, meaning in life and empowerment [3]. Within the CHIME framework, hope is connected to motivation for change. Our findings illustrate how processes related to musicking and music therapy lead to experiences of motivation and mastery that in turn impact identity in constructive ways and foster social belonging. Thus, our findings may contribute nuanced understandings of the interlocking processes that facilitate personal and social recovery in people with co-occurring ADHD and SUD.

Our findings demonstrate that it is important to understand the impulsivity, hyperactivity and inattention (IHI) that accompany ADHD in context, not just as isolated and unidimensional symptoms. ADHD is most often conceptualized as located within the individual and existing independent of context and time [13]. Participants experienced ADHD as a life-long process of coping, one for which they had developed tools including use of medication (prescribed or illicit), learning to pause and think through things, and using music to regulate energy, feelings, and thoughts. Their experience of ADHD and their corresponding coping tools were linked directly to the contexts in which they found themselves, and the tool of music emerged as relevant across many contexts. Participants learned to direct their energy, promote attention and use their spontaneity as a strength within music interactions with others in a variety of contexts. As such, music therapy served as the kind of flexible, cross-contextual tool that Young & Woodhouse [18] call for to make meaningful change in substance use related patterns.

Interestingly, our participants reported that engaging in music did not stimulate substance use craving. Instead, some participants described how engaging in making music and reflecting upon it helped to focus thoughts on constructive and health-promoting aspects, instead of on a desire to use substances. Thus, the experiences described by our participants may align more with healthy uses of music than with unhealthy uses [43], and may help explain findings from experimental research that music therapy reduces substance use craving [27].

### Critical reflection on the current study

The findings presented represent the experiences of eight unique people, who differ in terms of gender, language of origin, music history, phase of treatment and length of experience with music therapy among other aspects. These findings augment our understandings of how music may be a resource for people with ADHD and SUD. We have tried to trace when participants spoke of music therapy versus when they spoke of engagement in music in everyday life, but in some cases it was not possible to distinguish the two. Thus, a limitation of this study is that we are not able to completely differentiate the experience of music therapy from that of music in everyday life [44] in all aspects of the findings. The experiences described in this article reflect the relatively short time frame of the study and follow-up with service users in post-treatment phases is indicated. Our findings suggest that it was easier for some participants to learn to adapt to and engage in healthy uses of music in everyday life outside of treatment settings, while for others, the threshold for engagement was too high. Thus, it is important to explore longer-term impact of positive experiences of music and music therapy in people with co-occurring SUD and ADHD. Finally, it is important to acknowledge that our analysis, findings and conclusions reflect the complement of our theoretical and disciplinary orientations. The resource-oriented philosophy of the primary data analyst may have led to us noticing and emphasizing resources within the findings.

### Implications for research and practice

Our findings highlight the importance of using participatory, collaborative and resource-oriented forms of treatment to promote engagement and motivation in substance use treatment for people with ADHD. Participants’ freedom to choose what they do in music therapy directly impacted their motivation and made it more likely they would experience mastery. Our participants’ experiences suggest that music therapy promotes positive changes that align with recommended aims of psychotherapeutic approaches for adults with ADHD and SUD, namely, constructively managing feelings, modifying destructive patterns of thought and behavior, and fostering motivation for developing healthier lifestyles [18].

Transitional periods are particularly vulnerable for people with substance use problems [45], and our findings suggest that access to music making spaces and music groups with a focus on rehearsing and/or performing can offer important support and structure during transitional periods. Thus, we recommend systematic continuity of the music therapy offer to span transitional periods, so that users can be “knit” into community through music and music therapy.

This study addresses identified knowledge gaps in research by exploring user perspectives [29] of music therapy with a resource-oriented theoretical orientation [26, 27] as a non-pharmalogical supportive approach for co-occurring ADHD and SUD [19]. We recommend future research on mechanisms underlying the impact of music therapy on regulation of IHI, which could inform more tailored practice of music therapy.

## Conclusions

Service user perspectives are crucial for informing developments in health service provision. In this study, we centered the voices and perspectives of adults with co-occurring ADHD and substance use problems, to understand how they experience music and music therapy. Our findings suggest that active forms of engaging in music and music therapy enabled experiences of both motivation and mastery that ultimately afforded social belonging. Our participants’ nuanced descriptions illustrate the importance of motivation for them, and how music therapy can contribute to motivation in substance use treatment. In particular, the context surrounding musicking, adaptations from the music therapist, and social affordances of such musicking contributed to pleasure, mastery, participation, development of identity and social belonging, which in interaction generated motivation. This health-promoting process was simultaneously personal, social and situated. Participants used music and music therapy to regulate emotions, energy, concentration and thoughts, that served as pre-requisites for fuller participation in social communities. Musicking became a means of establishing drug-free identity and fellowship for several participants, and was a way to steer and master energy related to impulsivity, hyperactivity and inattention. Experiencing mastery and self-development in music therapy increased motivation for continuing substance use treatment for some participants. Our findings demonstrate social and personal change processes that take place in and around music therapy, and illustrate how music therapy can provide a motivating and inclusive therapeutic option for adults who desire a broad range of expression.

### Electronic supplementary material

Below is the link to the electronic supplementary material.

Supplementary Material 1

## Data Availability

The datasets generated and analyzed during this study are not publicly available due to the risk of compromising individual privacy, but are available from the corresponding author on reasonable request.

## Footnotes

1 Referring to Christopher Small’s (1998) articulation of *musicking*, where music is conceived of as activity, not object. [23] Small C. Musicking: The Meanings of performing and listening. Hanover, NH: Wesleyan University Press; 1998.

2 In Norway, as in most other European countries, music therapy education is granted at the master’s level.

3 Gender-specific treatment was established at this facility in the 1990s. It is recommended by the Norwegian national guidelines for treatment and rehabilitation of substance use problems and addiction and is part of typical in-patient practice in Norway.

4 ICD-11 was taken into use by WHO member states starting January 1, 2022.

5 We have chosen to use non-binary pronouns.

6 When it was possible to trace a user experience specifically to either “music (in everyday life)” or to “music therapy,” we have intentionally used those words differentially to indicate such.

7 Playing rock or other forms of popular music in small groups facilitated by one or more music therapists.
