# Supplementary material for: Steering the energy with music: hermeneutic phenomenological study of user perspectives of music and music therapy for co-occurring ADHD and substance use problems
Source: Subst Abuse Treat Prev Policy. 2024 Jan 23;19:10. doi: 10.1186/s13011-024-00594-x (PMC10807156; doi:10.1186/s13011-024-00594-x)
Supplement: Supplementary file 1 — Supplementary Material 1 [file 13011_2024_594_MOESM1_ESM.docx]

**Appendix**

**Interview Guide** (translated from Norwegian)

Interview completed by:

Interviewee code:

Date of interview:

**Background information**

Gender:

Age category (18–25 years, 26–40 years, 40+ years):

Pharmacological treatment: yes/no

Length of participation in music therapy (2–3 months, 3–6 months, 6 months – 1 year, over 1 year):

Diagnosed with ADHD (3–6 months ago, 6 months – 1 year ago, 1–2 years ago, more than 2 years ago):

Status in treatment (inpatient treatment, outpatient treatment, concluded treatment):

Introductory question

1. Can you tell us a little about your relationship to music and music experiences before you began music therapy?

Main questions

1. Can you tell us about what led you to choose music therapy?
2. Can you tell us about your experiences with music therapy?
   1. What parts of the music therapy offer have you participated in?
      1. Group therapy (which groups)?
      2. Individual therapy (what focus)?
   2. Can you mention any positive experiences?
   3. Can you mention something you think has been challenging?
3. Can you tell us about how you experience having ADHD?
   1. Is the diagnosis ADHD something you’ve gotten used to (or is recognizable, accepted)?
   2. Which ADHD symptoms are/or have been most prominent? How has it changed over time (if it has changed)?
   3. Which treatment forms or personal coping strategies have you used?
4. Can you tell us about whether, and if applicable how, music therapy has been important for you (in your social recovery)?
   1. What significance has it, if applicable, had in relation to drug problems?
   2. What significance has it, if applicable, had in relation to ADHD symptoms? Has it impacted your mastery over ADHD symptoms (if so, which ones), or is it more like a process where the symptoms recede more to the background and resources and opportunities are discovered, or both?
   3. What significance has it had for your daily life, your everyday experience (socially and in other ways)?

Concluding questions

1. Is there anything you would like to add?
2. Do you have any suggestions for changes or improvements regarding the music therapy offer?
